# HB-EGF Plays a Pivotal Role in Mucosal Hyperplasia During Otitis Media Induced by a Viral Analog

**DOI:** 10.3389/fcimb.2022.823714

**Published:** 2022-02-23

**Authors:** Takashi Sakamoto, Kwang Pak, Eduardo Chavez, Allen F. Ryan, Arwa Kurabi

**Affiliations:** ^1^ Department of Surgery, School of Medicine, University of California, San Diego, CA, United States; ^2^ Department of Otolaryngology, School of Medicine, University of California, San Diego, CA, United States; ^3^ Department of Surgical Sciences, University of Tokyo, Tokyo, Japan; ^4^ Research Section, Veterans Affairs (VA) San Diego Healthcare System, La Jolla, CA, United States; ^5^ Department of Neurosciences, School of Medicine, University of California, San Diego, CA, United States

**Keywords:** hyperplasia, EGFR, poly(I:C), mucosal proliferation, heparin-binding epidermal growth factor

## Abstract

Otitis media (OM), the most common childhood illness, can be caused by bacterial and/or viral infection. Hyperplasia of the middle ear (ME) mucosa is an important component of OM that contributes to its deleterious sequelae. Our previous research revealed that ME mucosal hyperplasia in bacterially induced OM was associated with expression of the heparin-binding epidermal growth factor (HB-EGF) gene, and that HB-EGF induced the proliferation of ME mucosal explants in culture. We used single-cell RNA-Seq to identify ME cells that express *Hbegf* and related genes involved in mediating responses to this factor. To determine the degree to which a viral infection might induce mucosal hyperplasia, and to assess the role of HB-EGF in hyperplasia *in vivo*, we used, Poly(I:C) to simulate a ME viral infection, Western blotting to confirm ME protein expression, and a specific inhibitor to block the effects of HB-EGF during OM. Genes for HB-EGF and its receptor were expressed in the ME primarily by epithelial, stromal and endothelial cells. Poly(I:C) induced prominent ME mucosal hyperplasia, peaking two days after ME injection. Immunostaining revealed that cleavage of proHB-EGF into its soluble form (sHB-EGF) was strongly induced in response to Poly(I:C). Inhibition of the sHB-EGF receptor dramatically reduced the hyperplastic response of the mucosa. The results demonstrate that a synthetic analog of viral double-stranded RNA interaction can induce OM including a strong proliferative response of the ME mucosa, independent of bacteria. They also indicate that HB-EGF is the dominant growth factor responsible for ME mucosal hyperplasia *in vivo*.

## Introduction

Otitis media (OM), one of the most common childhood infections (Casselbrant et al., 1993), can be a serious disease. In the US, OM causes more pediatrician visits, antibiotic prescriptions, and surgeries than any other condition for children under 5 years of age (Rovers, 2008). It is estimated to cost $5 billion per year in healthcare expenses and lost productivity (Teele et al., 1988; Rosenfeld and Bluestone, 2003). Chronic and recurrent OM occur in ~15% of children (Pichichero, 2016) and cause persistent hearing loss that has been linked to delays in speech (Friel-Patti and Finitzo, 1993), and language acquisition (Klausen et al., 2000), as well as deficits in learning (Williams and Jacobs, 2009).

OM is frequently polymicrobial, involving multiple bacterial genera/species and/or viruses (Ruohola et al., 2006; Marom et al., 2012). Clinical and experimental studies have shown that infection of the ME induces inflammatory responses, a major feature of which is ME mucosal hyperplasia. The mucosa of the resting ME consists primarily of a simple squamous epithelial monolayer and a minimal stroma, with a thickness of approximately 15-30 μm (Lim and Hussl, 1969; Lim, 1976). During OM, the mucosa rapidly transforms. The epithelium proliferates and remodels into a pseudostratified, respiratory-like epithelium that includes secretory, goblet, and ciliated cells. The stroma also proliferates, adding fibrocytes, connective tissue and neovascularization. The thickness of the mucosa can increase by tens to hundreds of μm in 2-3 days, reducing effective ME volume (Lim, 1979; Hernandez et al., 2008; Leichtle et al., 2010). Increased permeability of the enhanced vascular bed leads to infiltration of the ME lumen by fluid and leukocytes. Mucus and inflammatory mediators are also secreted into the lumen by ME cells (Ryan et al., 2020). Thus, hyperplasia of the ME mucosa is a major driver of inflammatory pathophysiology in OM.

While studies in animals have confirmed that bacterial infection induces mucosal hyperplasia, whether a purely viral infection also does so is less clear. The few experimental studies of virus injection directly into the ME have yielded contradictory results. Respiratory syncytial virus introduction induced very mild inflammation in the guinea pig ME (Berglund et al., 1966; Berglund, 1967), while influenza virus produced a vigorous response in the chinchilla ME, including mucosal hyperplasia similar to that in bacterial OM (Chung et al., 1993).

ME inflammation is generated in acute OM by the interaction of pathogen molecules with innate immune receptors, including the TOLL-like receptors (TLRs) and others (Muñoz-Wolf and Lavelle, 2016). These activate well-known inflammatory pathways leading to the expression of inflammatory mediators such as tumor necrosis factor alpha (TNFα) and interleukin 1 beta (IL-1β) (Kurabi et al., 2016). The mechanisms by which inflammation leads to ME mucosal hyperplasia are unclear. However, by analyzing the ME transcriptome in a mouse model of bacterial OM, we previously found that heparin-binding epidermal growth factor (HB-EGF) is up-regulated with kinetics suggesting a possible role in mucosal growth (Hernandez et al., 2015). Moreover, it was the only up-regulated growth factor capable of strongly stimulating ME mucosal explant expansion in culture (Suzukawa et al., 2014). It remains to be established whether HB-EGF mediates ME mucosal hyperplasia *in vivo*.

HB-EGF is a member of the EGF family of growth factors. It is expressed as a membrane-anchored glycoprotein pro-form (proHB-EGF). Various stimuli, including pro-inflammatory cytokines such as TNFα and IL-1β (Yoshizumi et al., 1992; Dluz et al., 1993), induce proteolytic processing of proHB-EGF to release a mature soluble peptide (sHB-EGF). The soluble form can interact with two EGF receptors, EGFR and ERBB4, in a paracrine and/or autocrine manner to induce responses. sHB-EGF binds to EGF receptors with higher affinity than EGF. These include cellular proliferation and differentiation leading to tissue growth (Dao et al., 2018). Which cells in the ME produce HB-EGF or its receptors is also unknown.

The innate immune response to viruses is distinct from that to bacteria, involving different pathogen receptors. In viral infection, viruses can produce double-stranded RNA (dsRNA) during replication within infected cells (Weber et al., 2006). Host cells recognize double-stranded RNA primarily *via* Toll-like receptor 3 (TLR3) (Matsumoto and Seya, 2008), as well as the nucleic acid receptors retinoic-acid-inducible gene I (RIG-I) and melanoma-differentiation-associated gene 5 (MDA-5) (Yoneyama et al., 2004; Gitlin et al., 2006). Stimulation of TLR3, followed by interaction with the adaptor protein TRIF, or of RIG-1 or MDA5, increases the release of type-I interferons and activates the nuclear factor (NF)-κB pathway leading to inflammatory cytokine production, as a host defense system (Jacobs and Langland, 1996; Matsumoto and Seya, 2008). In the current study Polyinosinic-polycytidylic acid [Poly(I:C)] was employed to mimic viral challenge. Poly(I:C) is a potent agonist of TLR3, MDA5 and RIG-1 (Jensen and Thomsen, 2012) and an established model of viral infection (Miyamoto et al., 2004; Matsumoto and Seya, 2008). In other tissues, exposure to poly(I:C) upregulates the expression of both viral innate immune signaling and effector genes (Li et al., 2012; Parvizi et al., 2012). Whether activation of viral innate immunity in the ME leads to mucosal hyperplasia, and if so whether this involves HB-EGF, remains to be clarified.

Herein, we mined single-cell RNA-Seq (scRNA-Seq) databases of the mouse ME before and after bacterial infection, to identify ME cells that produce HB-EGF and its receptor mRNAs during OM. We then tested the hypothesis that a synthetic analog of viral double-stranded RNA can induce OM that includes mucosal hyperplasia, and that this *in vivo* hyperplasia is mediated by HB-EGF.

## Materials and Methods

### Animals

Studies were conducted using C57BL/6 mice of both sexes, and male Sprague-Dawley rats (60-90 days for each species). Female rats were reserved for breeding, as there are no data suggesting differences in OM pathogenesis due to sex, although incidence is higher in human males (Kvestad et al., 2004). Mice were employed to take advantage of their well-defined genetics. Rats were used for inhibitor studies because their ME volume (50 μl) is much greater than the mouse (5 μl), making these studies more practical. All experiments were performed to National Institutes of Health guidelines and approved by VA San Diego Medical Center IACUC (protocol 13-031 for mouse and 13-032 for rat).

### Surgical Procedure

For scRNA-Seq, mouse otic bullae were exposed *via* a ventral approach under deep anesthesia (ketamine 50 mg/kg, xylazine 1 mg/kg, acepromazine 5 mg/kg in.05 ml, IP) as previously described in Melhus and Ryan (2000). A small hole was made using a 23-gauge needle. MEs were injected with 5x10^4^ nontypeable *Haemophilus influenzae* (NTHi) CFUs in 5 μl of saline. During surgical recovery animals were monitored carefully, placed on a warming pad, and given buprenorphine (0.05 mg/kg) through s.c. injection to limit post-operative discomfort. For Poly(I:C) studies, rat MEs were similarly exposed and 500 μg of Poly(I:C) dissolved in 40 μl of sterile saline was injected. Control animals for both species were injected with saline alone.

### Single-Cell RNA-Seq

The expression of the *Hbegf*, *Egf*, *Egfr* and *Erbb4* genes in individual cell types of the ME was assessed in an existing murine scRNA-Seq dataset of normal ME (Ryan et al., 2020) and also in data from MEs inoculated 24 hours previously with NTHi. Twenty-four hours was chosen to precede protein and cellular reactions which peaked at 2 days after Poly(I:C) injection. To obtain the data, three groups of six normal or six infected young adults C57BL/6 mice were sacrificed, and the ME tissue and luminal contents harvested from the 12 MEs of each group. Briefly, tissue was enzymatically digested and dissociated into single-cell preparations. Single-cell libraries were prepared using the Chromium Controller (10X Genomics, Pleasanton, CA) according to the manufacturers’ instructions and sequenced on an Illumina HiSeq 2500 (Illumina, San Diego). Reads were aligned to a murine reference genome and subjected to principal component analysis (PCA) clustering. Cell types identified based on well-recognized marker genes. Expression of *Hbegf*, Egf, *Egfr* and *Erbb4* mRNA unique molecular identifiers (UMIs) for the various cell type clusters was visualized using the cLoupe program (10X Genomics) cluster and violin plot displays. More detailed methods are available in Ryan et al. (2020).

### Measurement of ME Mucosal Thickness in Poly(I:C) OM

Six rat otic bullae were harvested from 3 rats at 1, 2, 3, 4, or 5 days after injection of Poly(I:C). The bullae were immersed in 4% paraformaldehyde (PFA), post-fixed in PFA for 2 days and subsequently decalcified in 10% EDTA with 4% PFA for 1 week. The tissue was paraffin-embedded, sectioned at 8 μm and mounted on silane-coated slides. Deparaffinized and hydrated sections were stained with hematoxylin and eosin (H&E). Mucosal thickness was measured morphometrically (FSX-BSW, Olympus) in three sections through the middle of the ME cavity, at six standardized locations, and the values averaged for each ME.

### Immunostaining

Deparaffinized and hydrated sections of control rat MEs, and Poly(I:C)-injected MEs harvested 2 days later, were heated in citrate solution (pH 6.0) for antigen retrieval. After quenching endogenous peroxidases, sections were incubated with anti-HB-EGF antibody (rabbit polyclonal, 1:200, Bioss) that recognizes both forms of the factor for 24 hours. Immunoreaction was detected by ImmPRESS mouse/anti-rabbit secondary antibody (Vector Laboratories) developed with 3, 3’-diaminobenzidine (DAB) chromogen (Vector Laboratories). Slides were counterstained only with hematoxylin.

### Western Blot

Rat ME mucosae from 4 MEs were harvested on days 1, 2, 3, 4, or 5 after injection of Poly(I:C) and homogenized in protein extraction buffer: 500 μl of T-PER lysis buffer (Pierce, Rockford, IL) supplemented with protease inhibitors (Roche, Indianapolis, IN) and sonicated briefly on ice. Three independent samples, from 4 MEs each, were generated at each time point. Protein concentrations were measured using a NanoDrop 2000 (Thermo Scientific). Ten μg of protein in 25 μl was loaded in each well of Bis-Tris Mini Gels 4-12% (Life Technologies) and electrophoresed. Proteins were electrotransferred to polyvinylidene difluoride membranes (PVDF) (Bio-Rad). PVDF membranes were probed with anti-β-actin antibody (mouse monoclonal, 1:10000, BD Transduction Laboratories) and the antibody against HB-EGF (rabbit, polyclonal, 1:3000, Bioss) that recognizes both forms of the factor, with which sHB-EGF can be distinguished from proHB-EGF (18.7 kDa) by its lower molecular weight (9.7 kDa). Membranes were incubated with horseradish peroxidase-conjugated secondary antibodies (anti-mouse antibody for β-actin, 1:20000) (anti-rabbit antibody for HB-EGF, 1:10000) and visualized with chemiluminescent detection (GE Healthcare). Light emission was captured with autoradiography film. Labeled band molecular weight was determined by comparison with a standard molecular weight ladder. The intensity of the bands corresponding to sHB-EGF and β-actin were quantified using ImageJ software and sHB-EGF band intensity was β-actin normalized. Values for the three independent samples at each time point were averaged.

### HB-EGF Inhibition

Diphtheria toxin uses membrane-bound proHB-EGF as its receptor, mediated by an EGF binding domain in the toxin molecule (Mitamura et al., 1995). CRM197 is a detoxified mutant of diphtheria toxin. It specifically and irreversibly binds the EGF domain of sHB-EGF, preventing its interaction with EGF receptors (Miyamoto et al., 2004). EC50 in reducing the proliferation of cancer cells *in vitro* is ~3 μg/ml (Tang et al., 2012).

After confirmation of the time of maximal mucosal thickness induced by poly(I:C) injection (2 days), the mucosal thickness was evaluated for the following six groups of rats. Group 1 MEs were injected with 40 μl sterile saline. Group 2 MEs were injected with 500 μg of Poly(I:C) in saline. Group 3 was injected with 500 μg of Poly(I:C) plus 4 μg (100 μg/ml) of CRM197 (Reagent Proteins). Groups 4-6 were injected with Poly(I:C) plus 10, 1 or 0.1 μg/ml, respectively, of CRM197.

### Statistical Analysis

Statistical analyses were performed using GraphPad Prism 5 software. Data are reported as means ± standard errors of the means (SEM). Two-way ANOVA with Bonferroni correction for multiple comparisons was performed on measures of mucosal thickness. Data normality was evaluated by using the D’Agostino-Pearson omnibus test. Left and right ears in each mouse were considered to be independent of each other, as previously discussed in detail (Ebmeyer et al., 2005), and therefore were analyzed independently. Western blot relative band intensities were analyzed by Kruskal-Wallis nonparametric ANOVA, also with Bonferroni correction.

## Results

### Expression of *Hbegf*, *Egfr* and *Erbb4* Genes in Cells of the Murine ME Before and After Bacterial Infection

**Figure 1:**
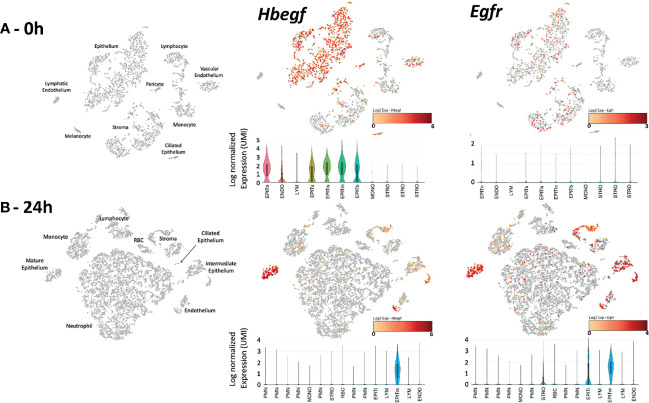
Expression of *Hbegf* and *Egfr* genes in the different cell types of the normal **(A-0h)** and infected **(B-24h)** mouse ME. Clustering of ME cells (in gray) and their identification is displayed in PCA plots, with cells expressing of *Hbegf* and *Egfr* shown in a continuum from pale organ to red indicating relative expression level according to each scale. Violin plots provide quantitative analysis, with height showing relative intensity of expression and width indicating the number of cells (UMIs) at that expression level. **(A-0h)** *Hbegf* is expressed primarily by five clusters of ME epithelial cells representing recognized ME subtypes including basal (EPITb), secretory (EPITs), intermediate secretory (EPITis), intermediate non-secretory (EPITin) and non-secretory including ciliated (EPITn) (Ryan et al., 2020), by vascular endothelial cells (ENDO) and by small subsets of other cells. *Egfr* is expressed by subsets of all epithelial and stromal (STRO) cell clusters. **(B-24h)** Twenty-four hours after NTHi infection, large numbers of infiltrating neutrophils (PMN) in six clusters, monocytes (MONO) and lymphocytes (LYM) dominate the ME, with structural cell types in smaller proportions and epithelial cells in only two clusters: mature (EPITm) and intermediate (EPITi). The different PMN subtypes reflect the increasingly recognized and as yet poorly understood diversity of neutrophils (e.g. Hesselink et al., 2019). As in the normal ME, *Hbegf* is expressed primarily by epithelial and vascular endothelial cells. Normalized gene expression by epithelial cells was higher than in that in the uninfected ME (mean of 2.42 log_2_ UMIs versus 1.58 log_2_). *Egfr* mRNA is present in most epithelial and stromal cells, but also in vascular endothelial cells. Epithelial cell (0.86 log_2_ UMIs) and stromal cell (0.50 log_2_) expression was higher than that in the uninfected ME (0.11 log_2_ for both) In PCA plot cells, the color orange indicates detectable expression of the gene.

Single-cell RNA-Seq was employed to determine whether genes related to HB-EGF signaling are expressed in the ME, and to identify the cells involved. In the untreated ME (**Figure 1A**-0h) *Hbegf* was expressed strongly by most cells in five epithelial cell clusters and a substantial minority of vascular endothelial cells, but also by small numbers of other ME cell types. *Egfr* was expressed by a significant minority of epithelial and stromal cells, but not by other cell types. *Egf* mRNA was observed in only a few scattered epithelial, stromal and leukocytic cells and *Erbb4* mRNA in only a few epithelial cells (not shown). At 24 hours after NTHi inoculation (**Figure 1B**), *Hbegf* was observed in all mature epithelial cells, and in a substantial minority of stromal, intermediate epithelial and endothelial cells. Smaller numbers of cells in leukocyte clusters also expressed this gene. *Egfr* mRNA was also observed at high levels in all mature epithelial cells, in the majority of stromal, intermediate epithelial and endothelial cells, and in much small minorities of other cell types. *Egf* mRNA was observed in only a few scattered epithelial, stromal and leukocytic cells and *Erbb4* mRNA in only two epithelial cells (not shown).

### Poly(I:C) Induces ME Mucosal Hyperplasia

**Figure 2:**
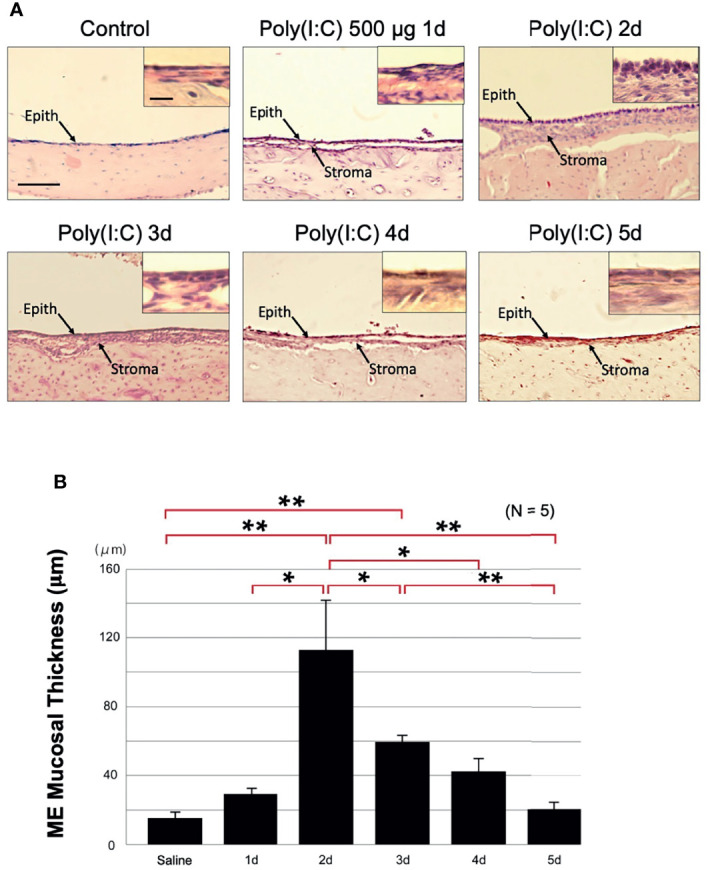
Poly(I:C) enhances rat ME mucosal thickness. **(A)** Representative microphotographs of rat ME mucosa are shown. Control ME is saline-injected at 2 days. Mucosal thickness is maximal 2 days after administration of 500 μg of Poly(I:C) then gradually recovers, becoming similar to the control on day 5. Scale bars: 100 μm for main image, 25 μm for insert **(B)** Morphometrically measured mucosal thickness confirms increase to a maximum at day 2 after administration of Poly(I:C), then a gradual decrease to the control level by day 5. N refers to number of MEs. *p < 0.05; **p < 0.01.

To determine whether a model of viral infection would induce OM as evidenced by mucosal hyperplasia, 500 μg of the viral analog Poly(I:C) was injected into the rat ME. This resulted in acute hyperplasia of the ME mucosa (p < 0.01). Changes in mucosal thickness from normal (16 μm [SEM = 3 μm]) primarily reflected expansion of the mucosal stroma. The mucosal epithelium added somewhat fewer cells, but the epithelial cells assumed a more cuboidal morphology (**Figure 2A**). Mucosal thickness peaked at 2 days after injection at 112 μm (SEM = 27 μm), and then gradually recovered to near normal by day 5 (**Figure 2B**). Compared to bacterial OM (Hernandez et al., 2008; Leichtle et al., 2012), fewer leukocytes were observed in the ME lumen during poly(I:C) OM.

### Poly(I:C) Increases ME Processing of HB-EGF Into Its Soluble Form

**Figure 3:**
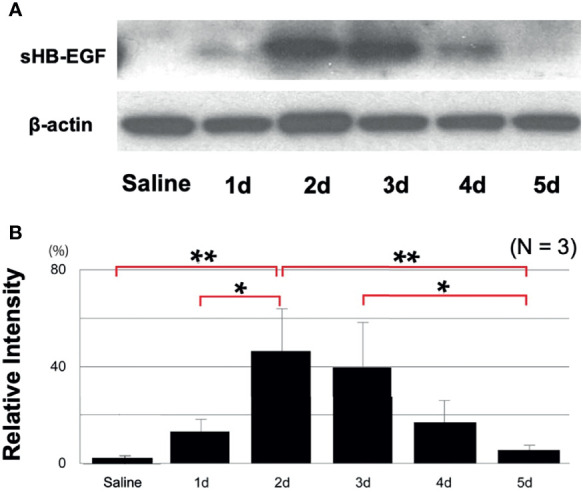
Western blotting demonstrates HB-EGF processing during OM. **(A)** Representative blots for rat MEs are shown. The processed form, sHB-EGF, was distinguished from proHB-EGF by its lower molecular weight, **(B)** Relative band intensity quantification confirms that sHB-EGF increases to a maximum 2 days after administration of 500 μg of Poly(I:C), then progressively decreases. Saline control mucosa at 2 days shows a low sHB-EGF level. N refers to number of samples. *p < 0.05, **p < 0.01.

While our gene expression data confirm ME expression of the *Hbegf* gene during OM, they do not determine whether it is processed into the soluble form that activates EGF receptors. Western blotting of rat ME tissue with an antibody against HB-EGF (**Figure 3A**) showed essentially none of the soluble form prior to poly(I:C) injection (relative band intensity 2%, SEM = 1%) and a barely detectable band (12%, SEM = 9%) on day 1. This was followed by a strong increase that peaked at 2 days after injection (47%, SEM 19%; p <.01), a more than 20-fold increase), and then gradually decreased to almost undetectable at Day 5. Relative sHB-EGF band intensity, shown quantitatively in **Figure 3B**, was highly significant when compared to pre-injection levels. While proHB-EGF was not analyzed by Western, our scRNA-Seq data indicate that expression levels of the *Hbegf* gene by ME epithelial cells increased at 24 hours after bacterial inoculation.

### An Inhibitor of sHB-EGF Interaction With EGF Receptors Decreases Mucosal Hyperplasia During Poly(I:C)-Induced OM

**Figure 4:**
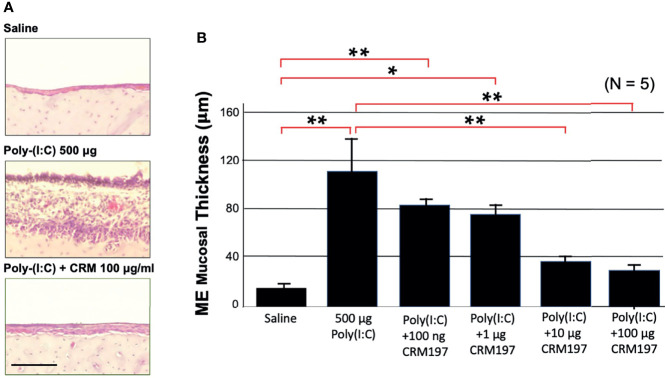
An HB-EGF inhibitor decreases mucosal thickness during OM. Poly(I:C)-induced hyperplasia of rat ME mucosa was inhibited by co-administration of CRM197. **(A)** Representative microphotographs are shown on day 2. Scale bar = 100 μm. **(B)** Mean thickness of saline ME mucosa versus 500 ug of Poly(I:C), without or with various levels of CRM197 on day 2. N refers to number of MEs. *p < 0.05; **p < 0.01.

To determine the degree to which sHB-EGF might contribute to mucosal hyperplasia, we used a specific EGFR inhibitor. **Figure 4A** shows representative photomicrographs of the mucosa from a control rat ME, and a ME two days after injection of poly(I:C) or poly(I:C) plus 100 μg of CRM197, while **Figure 4B** provides a quantitative analysis of mucosal thickness observed with various CRM197 dosages. The increase in thickness of the mucosa observed after poly(I:C) injection, 112 μm (SEM = 27 μm) was largely absent in the ME injected with poly(I:C) plus 100 μg CRM197, 13 μm, (SEM = 4 μm). This difference was highly significant (p < 0.01). Significant reduction was also seen at a CRM197 dosage of 10 μg (p < 0.01).

### Expression of HB-EGF Is Not Affected by CRM197

**Figure 5:**
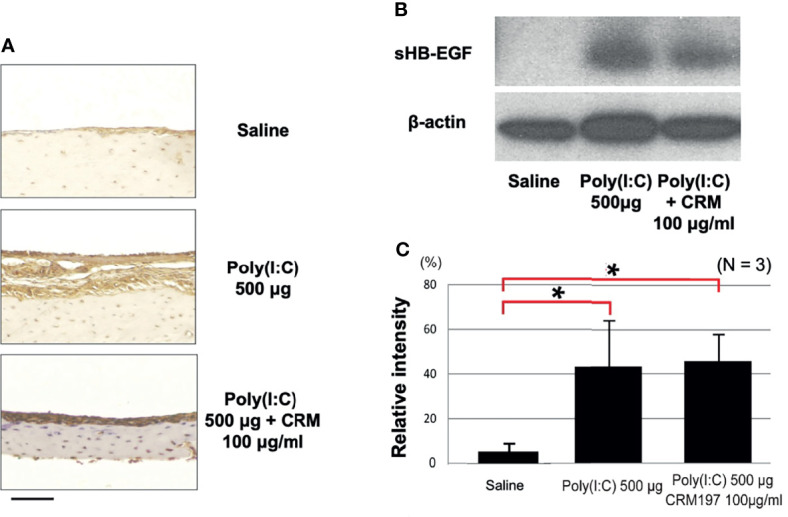
CRM197 does not inhibit HB-EGF expression or processing. **(A)** Representative microphotographs of immunostaining with a pan anti-HB-EGF antibody are shown. HB-EGF was expressed in rat ME mucosa in all 3 groups on day 2. **(B)** Representative Western blot sHB-EGF bands for rat ME, 2 days after saline injection or Poly(I:C) administration with or without CRM197. **(C)** Quantitative analysis showed that sHB-EGF was low in the saline samples but was increased by both Poly(I:C) and Poly(I:C) plus CRM197. N refers to number of samples. *p < 0.05.

CRM197 is a receptor inhibitor, but given the potential for receptor feedback, we used immunohistochemistry to determine whether the inhibitor also influenced the amount of HB-EGF expressed in the ME. **Figure 5** shows representative images of the rat ME mucosa immunostained with an antibody against both forms of HB-EGF. Strong staining was observed in the control ME, the ME injected with poly(I:C) or the ME injected with poly(I:C) plus CRM197, indicating minimal effects of the inhibitor on HB-EGF production.

### Processing of HB-EGF Into Its Soluble Form Is Not Affected by CRM197

Receptor feedback might also have influenced the processing of HB-EGF into sHB-EGF. **Figure 4** shows Western blotting with an anti-HB-EGF antibody of bands corresponding to the weight of sHB-EGF, in normal MEs and in MEs injected with poly(I:C) or CRM197 plus poly(I:C). The level of sHB-EGF was low in controls (mean relative intensity 5%, SEM 8%), and increased after Poly(I:C) (43%, SEM 21%; p <.01), a more than 8-fold difference. Moreover, the inhibitor did not affect the level of processed HB-EGF (44%, SEM 16%), indicating no effect on processing.

## Discussion

Several novel observations were made in this study. ME administration of Poly(I:C) induced OM that included mucosal hyperplasia and leukocyte infiltration. The ME cells of origin of HB-EGF and its primary receptor were identified, both for the normal ME and during OM. Finally, inhibition of HB-EGF signaling reduced mucosal hyperplasia to a level not significantly different from that in the uninfected ME.

The fact that Poly(I:C) induced the major pathophysiologic elements of OM in the rat ME is perhaps not surprising, since dsRNA interaction with TLR3 and other nucleic acid receptors including MDA5 and RIG-1 induces not only interferons, but also many of the same inflammatory mediators produced by bacterial infection (Jensen and Thomsen, 2012). However, this observation indicates that purely viral OM would likely induce significant ME pathology that was resistant to antibiotic therapy. This conclusion is supported by the results of Chung et al. (1993) that influenza virus inoculation of the ME produced hyperplasia, but not by the results of Berglund et al. (1966; Berglund, 1967) that RSV injection produced minimal ME mucosal growth.

ScRNA-Seq determined that *Hbegf* mRNA is produced primarily by subsets of ME epithelial, endothelial, and stromal cells, as is *Egf*r mRNA. Moreover, as can be seen in **Figure 1**, the cells producing of *Hbegf* and *Egfr* mRNA are very closely matched. This suggests that HB-EGF likely acts in an autocrine manner in the ME. It should be noted that the scRNA-Seq data were generated by bacterial infection of the mouse ME, not Poly(I:C) injection. These data were used since we had an existing database. However, we acknowledge that that Poly(I:C) might induce different cellular responses in the rat ME.

ScRNA-Seq and immunostaining demonstrate that HB-EGF is present in the normal rat ME but increases during Poly(I:C)-induced OM. Increased expression of HB-EGF in the ME by Poly(I:C) is consistent with the induction and processing of this growth factor during the development of poly(I:C)-induced lupus nephritis (Triantafyllopoulou et al., 2010). It also suggests that HB-EGF production is a feature of the response to viral infection. We could find no studies evaluating HB-EGF production during viral infection, although Poly I:C has been reported to up-regulate HG-EGF in kidney cells (Triantafyllopoulou et al., 2010). However, Ras activation can induce expression of the HB-EGF gene (Benn and Schneider, 1994; McCarthy et al., 1995), and many viruses are known to activate Ras signaling (e.g. Schreiber et al., 2020). In the ME, processing into sHB-EGF only appears to occur only after activation of dsRNA receptors by Poly(I:C). It seems highly likely that stimulation of TLR3/TRIF, RIG1 and/or MDA5 in the ME leads to the expression/activation of one or more metalloproteinases, such as MMP9 (Royer et al., 2017), ADAM 9, 10, 12 or 17 (Nanba et al., 2003), that can cleave proHB-EGF and release sHB-EGF to interact with EGF receptors.

The highly similar time courses of ME sHB-EGF level and mucosal thickness change after Poly(I:C) injection implicates a causal relationship between the two. Moreover, the scRNA-Seq data indicate that EGFR, a receptor for sHB-EGF, is expressed by both epithelial and stromal cells, suggesting that both major components of the mucosa can be stimulated. sHB-EGF interaction with EGFR is thus positioned to be a major driver of ME mucosal hyperplasia induction. This was confirmed in the inhibition study, in which the specific HB-EGF receptor inhibitor CRM197 essentially abolished ME mucosal hyperplasia induced by Poly(I:C), reducing growth of both the epithelium and the stroma.

Western blotting revealed that active sHB-EGF was elevated in both Poly(I:C), and Poly(I:C) + CRM197 MEs, compared to control MEs. This is consistent with the inhibitory mechanism of CRM197, which combines with sHB-EGF and prevents binding to EGF receptors (Miyamoto et al., 2004; Yagi et al., 2009) rather than inhibiting HB-EGF processing. It also demonstrates that the procedures used in this study did not reduce HB-EGF expression or its processing due to receptor-mediated feedback.

Taken together, the results of this study provide convincing evidence that sHB-EGF interaction with its EGFR receptor plays a pivotal role in ME mucosal hyperplasia in viral OM *in vivo*. Since many ME infections include viral and bacterial co-infection (Marom et al., 2012), HB-EGF inhibitors could be a useful adjunctive therapy to antibiotics treatment for OM that is unresponsive to antibiotics. They could reduce sequelae such as mucus production and ME volume reduction due to viral co-infectants and/or residual inflammatory bacterial components.

Reduction of pathology in chronic OM could be of value in the developing world, and in vulnerable populations of the developed world, where OM has more serious consequences. It is estimated by the WHO that undertreated OM results in 30,000 annual childhood deaths and one half of the world’s burden of handicapping hearing loss (WHO, 2004; WHO, 2020; Leach et al., 2020), due to progression from chronic OM to chronic suppurative OM with tympanic membrane perforation. This allows access to the ME by more pathogenic bacteria that reside in the external auditory canal, including *Pseudomonas aeruginosa* and *Staphylococcus aureus*. These more invasive and destructive pathogens increase damage to the middle and inner ears, leading to permanent hearing loss and risk of progression to meningitis. A treatment that in combination with antibiotics could reduce the pathogenesis of chronic OM might reduce this incidence of this progression and concomitant disease severity.

## Data Availability Statement

The data presented in the study are deposited in the Science Data Bank repository, accession number: 31253.11.sciencedb.01487 DOI: 10.11922/sciencedb.01487.

## Ethics Statement

The animal study was reviewed and approved by Institutional Animal Care and Use Committee of the VA San Diego Medical Center.

## Author Contributions 

AK and AR conceived the study. TS, KP, and EC performed experiments. AK wrote the primary manuscript text and prepared the figures. All authors contributed to the article and approved the submitted version.

## Funding

Supported by NIH/NIDCD grants DC000129, DC006279,DC014801 and VA Research Service grant BX001205.

## Conflict of Interest

AR is a co-founder of Otonomy Inc., serves as a member of the Scientific Advisory Board, and holds an equity position in the company. The UCSD Committee on Conflict of Interest has approved this relationship. Otonomy, Inc. played no part in the research reported here.

The remaining authors declare that the research was conducted in the absence of any commercial or financial relationships that could be construed as a potential conflict of interest.

## Publisher’s Note

All claims expressed in this article are solely those of the authors and do not necessarily represent those of their affiliated organizations, or those of the publisher, the editors and the reviewers. Any product that may be evaluated in this article, or claim that may be made by its manufacturer, is not guaranteed or endorsed by the publisher.
